# Peer support to address mental health, social determinants of health, financial wellness, and reentry navigation

**DOI:** 10.3389/frhs.2026.1788228

**Published:** 2026-06-16

**Authors:** Chyrell D. Bellamy, Elizabeth H. Flanagan, Stephen Hoffler, Sophia Acevedo, Giancarlo Ocasio, Elizabeth Brisola, Kayla Barone, Razi Kitaneh, Kimberly D. Blackman, Bridgett Williamson, Patricia Benedict, Trevor Perry, Richard Youins, Tino Negron, Luz Ocasio, Anna Roca, Katherine Ponte, Annie Harper

**Affiliations:** 1 Yale Program for Recovery and Community Health, Yale School of Medicine, New Haven, CT, United States; 2 Department of Social Work, Southern Connecticut State University, New Haven, CT, United States

**Keywords:** financial wellness, incarceration, mental health, peer support, recovery

## Abstract

**Introduction:**

Incarceration in the United States disproportionately affects individuals with mental illness and substance use disorders and often exacerbates mental health conditions due to the trauma of arrest, separation, disrupted care, and stigma. Forensic Peer Support (FPS) can improve recovery outcomes by building unique trust and rapport that supports community reintegration and recovery outcomes.

**Method:**

This paper describes findings from a qualitative study of 22 participants who are a sub-sample of the larger Recovery Finance study, a multi-level community based participatory research study designed to reduce financial hardships of individuals involved in criminal justice with mental health challenges through system level interventions as well as an individual intervention (randomized control trial testing one-on-one financial capability support (FCS) versus FCS plus forensic peer support). The focus of this paper is the mental health and peer support experiences of the qualitative sub-sample.

**Results:**

Thematic analysis of in-depth qualitative interviews revealed the following themes: 1. The Impact of Incarceration, Trauma, and Mental Health, 2. Systemic Failings in Mental Health Care in Prison and Post-Release, 3. The Cycle of Financial Strain and Mental Health, and 4. The Unique Value of Shared Lived Experience through Peer Support. Results highlight the trauma and psychological impact of incarceration, systemic failures in treatment, financial stress as a dominant source of anxiety, often perpetuating cycles of poverty and criminal activity, and the critical role of supportive relationships, in particular FPS.

**Discussion:**

These findings point to the need for integrated models in which forensic peer support is embedded within financial wellness and reentry services.

## Introduction

### Incarceration, mental illness/SUD, and systemic barriers to reentry

The United States has one of the highest incarceration rates in the world, with approximately 530 individuals per 100,000 population incarcerated (1), a rate more than five times higher than other industrialized nations (2). Mass incarceration is recognized as a public health problem that contributes to significant racial and economic disparities within the criminal justice system (3, 4). Research has shown that Black, Latine and Native populations are much more likely to be incarcerated than White, non-Hispanic individuals, partly because of unfair treatment in policing and sentencing (5–7).

Furthermore, individuals from low-income communities are at a significantly higher risk of incarceration, often due to limited access to education, employment opportunities, and legal representation (8, 9). These structural inequalities create a cycle in which underrepresented populations are more likely to be criminalized and less likely to receive the support needed for successful reentry into society.

Compounding this issue is the historical criminalization of mental illness. Jails and prisons have become the nation's de facto mental health institutions after the closure of psychiatric hospital and inadequately funded community-based care left people without adequate treatment (10–13). Today, an estimated 44% of people in US jails and 37% in prisons have a mental illness, compared to 18% in the general population (14), and an estimated 63% of people in jail and 58% in prison have a substance use disorder (14). Despite these rates, 33% with a mental illness in state prisons and 66% in federal prisons report not receiving mental health treatment since admission (15), even though the trauma of arrest, familial separation, disrupted care, and pervasive stigma can worsen mental health conditions (16). These factors fuel the “revolving door” phenomenon, where untreated symptoms and systemic barriers increase the likelihood of recidivism, especially when mental illness remains untreated after release (10, 17).

Reentry into the community presents multiple challenges, including securing stable housing, employment, and financial stability, while rebuilding social supports (18). For individuals with mental health conditions, these hurdles are magnified by the simultaneous need to access treatment amidst stigma, poverty, and fragmented service systems (19). This population faces elevated risks of homelessness and unemployment, which are strongly associated with higher recidivism rates (20, 21). Individuals with mental illness are reincarcerated more frequently than those without, underscoring a critical gap in supportive reentry services (22, 23).

This study is grounded in the recognition that successful reentry is not solely a matter of addressing behavioral health needs but also requires attention to financial wellness as a critical and often overlooked social determinant of health. For individuals returning from incarceration, financial instability, including lack of income, debt, limited access to banking, and barriers to employment, interacts bidirectionally with mental health, exacerbating stress, undermining recovery, and increasing risk of recidivism. Peer support offers a uniquely positioned approach to address this intersection. Individuals with lived experience of incarceration and recovery are able to build trust, reduce stigma, and engage participants in conversations that extend beyond traditional service boundaries, including financial challenges, survival strategies, and structural barriers. In this way, peer support serves not only as a behavioral health intervention, but also as a bridge to financial capability, resource navigation, and long-term stability.

### Peer support and forensic peer support as a critical intervention

Peer support in the mental health field is an established, recovery-oriented practice that involves individuals with lived experience of mental illness and/or substance use recovery providing holistic, non-clinical support to others facing similar challenges (24, 25). In the United States, peer support is increasingly integral to the behavioral health landscape, with over 30,000 trained peer specialists and Medicaid reimbursement available in 43 states (26, 27). By leveraging shared experiential knowledge, peer specialists effectively foster hope, reduce stigma, and engage individuals who may distrust traditional clinical systems (25, 28). A growing, albeit heterogeneous, evidence base links peer support to positive outcomes, including reduced inpatient service use and improved quality of life and hope (22, 29, 30).

Forensic Peer Support (FPS) is a specialized peer support approach for justice-involved populations. FPS specialists possess lived experience with both mental health, substance use, or co-occurring challenges and the criminal justice system (31). This dual expertise allows them to build unique trust and rapport while providing practical assistance with community reintegration, accessing services, managing psychological and financial stressors, navigating probation and parole, and adhering to supervision mandates (31, 32). Emerging research indicates FPS can improve recovery outcomes, increase engagement with treatment and housing, and reduce recidivism (22, 33–35). Furthermore, serving as a peer specialist can itself be a recovery-promoting experience, fostering empowerment, purpose, and a positive identity beyond that of “patient” or “inmate” (36). Grounded in principles of hope, self-determination, and holistic wellness, FPS represents a promising, person-centered approach.

### The current study

**Figure 1:**
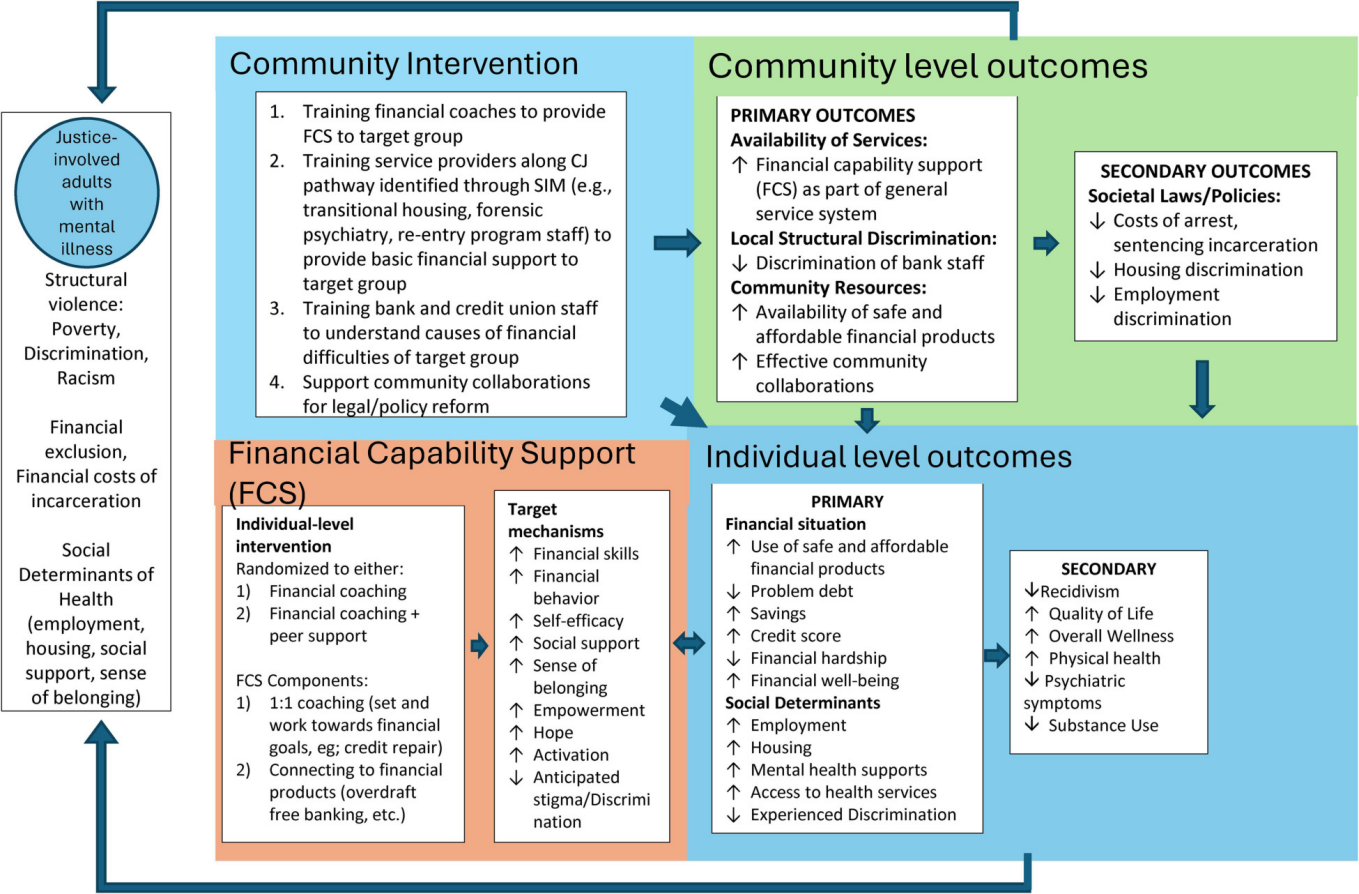
Conceptual model for the recovery finance study.

The Recovery Finance study is a multi-level intervention, utilizing a Community-Based Participatory Research (CBPR) framework, that is designed to reduce the financial hardships faced by justice-involved individuals with mental health challenges by directly responding to identified gaps in reentry support to address the critical yet under-addressed role of financial instability as a key social determinant of health (SDOH) (see conceptual model, Figure 1). The system-level intervention targets financial coaches, bank staff, service providers and community collaborations. The individual intervention tests the added value of peer support to financial coaching through a randomized control trial where half of the participants were randomly assigned to receive six months of one-on-one financial capability support (FCS) which includes coaching and help accessing safe and affordable bank accounts, while the other half received financial capability support plus FPS. FCS was provided by a trained financial coach working for an organization that provides supports to the reentry population. FPS was provided by peer supporters trained as recovery coaches or peer support specialists who in addition received forensic peer support training and training on the 8 dimensions of wellness, equipping them with additional skills to assist participants in addressing SDOH challenges. Peers received biweekly supervision and met to discuss participants, training technologies are used nationwide for peer supporters. Peers also co-authored this manuscript, in particular writing about their experience as peer supporters. The RCT tests the hypothesis that FPS would enhance outcomes by improving resource navigation, modeling recovery, and instilling hope through shared lived experience—a premise supported by our team's prior research on peer support's role in mitigating inequities. The Recovery Finance study is currently still in follow-up data collection.

Recovery Finance is the first study of its kind, to our knowledge, to integrate financial capability services and peer support in the context of a population who has both experienced incarceration and has mental health challenges. It is also innovative in its aims to not only provide support to individuals but also to change community level factors shaping financial well-being after incarceration, particularly through addressing financial institution practices.

The purpose of this paper is to examine how individuals with recent incarceration and mental health challenges experience the intersection of mental health, financial stress, and peer support within a financial wellness intervention, with particular attention to the role of peer support in addressing financial instability as part of recovery and reentry. Since Recovery Finance uses a mixed method (quantitative-qualitative), multi-layer (system and individual), CBPR approach for the larger study, we offer the baseline characteristics of the enrolled subsample in the RCT to provide context for the qualitative subsample examined here.

## Materials and method

This research was reviewed and approved and is monitored by the Yale Human Investigation Committee. Participants provided written informed consent to participate in this study.

### Participants and procedures

Participants were recruited from halfway houses, warming centers, community service providers, bus stops, public events, and word of mouth [especially through our consultants, Community Advisory Board (CAB) members, and already enrolled participants]. Potential participants were told about the study by staff, other clients, or our recruiters (e.g., peer support specialists, CAB members, research assistants) who visited or worked at relevant sites.

Participants self-identified that they wanted to participate. A total of 344 unique participants were screened for participation in the Recovery Finance intervention; 234 unique adults were enrolled. Inclusion/exclusion criteria were: being at least 18 years old, having a recent incarceration experience (prison or jail within the prior three years of enrolling in the study, no minimum length of time), self-identified mental health or substance use challenges, living in or used services in the greater area (i.e., county) of a mid-sized city in the northeastern United States, and being interested in receiving financial guidance. The inclusion criteria of three years post-incarceration was determined by our CAB, consultants, and forensic peer supporters based on their assessment that it can take considerable time for some people after release to be able to focus on their finances. From this pool of enrolled participants, a sub-sample of 22 participants was purposively selected for in-depth qualitative tracking. Selection of the qualitative sample aimed for diversity across randomization groups (financial capability support only vs. financial capability support + FPS) and baseline levels of financial strain. These 22 participants completed semi-structured qualitative interviews twice during the project period.

### Data collection and analysis

#### Qualitative data collection and analysis

The qualitative interview guide was drafted based on our conceptual model (see Figure 1) including questions about incarceration history, its impact on mental health and finances, current mental health and financial situation, and future hopes. We then refined the topics and questions in consultation with our CAB and consultants with lived experience (37) and pilot tested it. Our CAB members are people with lived experience of incarceration, mental health, and substance use challenges, who were recruited through our research team's contacts (from personal and prior professional/research work) and local re-entry support and advocacy organizations. At the time of writing, 22 baseline interviews and 15 follow-up interviews (conducted between 6 and 12 months) had been completed, and no participants had dropped out voluntarily (one had been reincarcerated). We had originally planned for a sub-sample of 18 participants but increased the number to 22 as we reviewed completed transcripts, both because we were concerned that we may not reach saturation across transcripts, given the diversity of participant experiences, and to mitigate possible attrition due to follow up/recidivism. We are confident that we ultimately reached saturation, acknowledging that each additional person interviewed inevitably adds unique elements to any qualitative dataset (38).

Participants were paid $40 for completing a qualitative interview, which lasted between 35 and 90 min. All interviews started with a description of the research goals, as part of the consent, and the interview introduced themselves and explained their role in the project. All but one interview was conducted in person (one by zoom) in a private room, at either a local public library or the research team office, and all were audio-recorded then transcribed using Trint software. Transcripts were verified for accuracy by research team members, who also wrote reflective memos after each interview to capture non-audio observations.

Interviews were conducted by a study principle investigator (PI), or research assistants trained by a PI. The interviewer team is racially and gender diverse and includes one person with personal experience of incarceration and several that identify with lived experience of mental illness; people who served as peer supporters for the intervention were not part of the interview or initial analysis team. One of the PIs is a trained cultural anthropologist with expertise in financial hardship and mental health, including among people previously incarcerated in this community, but has no personal experience of mental health or incarceration. She works closely with colleagues who are trained peers with such experience to inform her approach to her work, and to try to mitigate gaps in understanding due to her lack of personal experience.

We used a grounded theory approach primarily rooted in inductive strategies while incorporating deductive strategies, to analyze the qualitative data (39, 40). The qualitative analysis team, which included members of the interviewer team in addition to four additional people conducted open coding on a sub-sample of six transcripts (at least two people analyzing each transcript), meeting every two weeks over a two month period to compare and come to consensus on code labels and descriptions. We then integrated these inductively derived codes with deductive parent codes from our interview guide which included: incarceration experiences; mental health before, during and after incarceration; relationships and social supports; finances before, during and after incarceration; peer support; financial guidance from coach (these latter questions were only included in the follow-up interview guide to create a codebook).

For this paper, three analysts used NVivo software to code all transcript sections pertinent to mental health and peer support. One author (AH) reviewed all transcripts in full then compiled the relevant coded sections into draft themes and identified illustrative quotes from the transcripts (40, 41). The other analysts then reviewed and commented on the themes before they were finalized by AH. The findings presented below center participants’ experiences, supplemented by reflections from the peer supporters who worked on the project.

#### Quantitative data collection and analysis

Enrolled participants were asked to complete quantitative surveys at baseline, 6 months, and 12 months after baseline. Surveys were completed face-to-face with a trained research assistant unless the participant chose to answer the questions individually on a tablet.

Participants could choose to receive the interview in Spanish with a bilingual, bicultural research assistant. Participants were paid $40 for each interview they completed. For this analysis, only the baseline data for the qualitative subsample and a subset of the quantitative measures collected are reported.

The measures reported in this paper are as follows. The Patient Health Questionnaire-9 (PHQ-9) is a 9-item (item range 0–3), diagnostic tool for depression with higher ratings indicating more depression and with a summary score of 10 of higher indicating moderate depression (42). PROMIS SF v1.0 - Anxiety 8a is an 8-item, self-report questionnaire assessing the frequency of anxiety symptoms over the past 7 days using a 1–5 scale (“Never” to “Always”), where higher scores indicate more anxiety (43). The Posttraumatic Stress Disorder Checklist for DSM-5 (PCL-5) is a 20-item self-report questionnaire measuring PTSD symptoms ranging from 0 (“Not at all”) to 4 (“Extremely”), with a sum score of 33 indicating a probable diagnosis of PTSD (44). The Money and Mental Health Scale is a 9-item measure of the link between financial distress and psychological wellbeing where items range from 0 - “Not at all” to 4 - “Most of all of the time” (45).

The Adverse Childhood Experiences (ACEs) measure is a 10-item questionnaire with each question rated 0–1 (no-yes) assessing childhood trauma including abuse, neglect, and household dysfunction, with a score of 4 of higher indicating high risk for chronic diseases, mental illness, and social challenges in adulthood (46). The 8 Dimensions of Wellness measure is a 67-item measure with items ranging from “Never True” to “Always True” (range 1–4) of emotional, physical, social, spiritual, intellectual, environmental, occupational, and financial wellness and quality of life, with higher scores indicating more wellness (47). The Accountable Health Communities (AHC) Health-Related Social Needs (HRSN) Screening Tool is a 24-item tool developed by the Centers for Medicare & Medicaid Services (CMS) Center for Medicare and Medicaid Innovation (CMMI) to assess health-related social needs, with individual items having different ranges and higher scores indicating more health- related social needs (48),

### Reflections from recovery finance FPS peer supporters

As a final data source, the project's Forensic Peer Support specialists provided written reflections on their work, to ground the qualitative and quantitative findings in practice and to exemplify the application of peer support within the Recovery Finance intervention.

## Results

### Demographic and mental health characteristics

Demographic and mental health characteristics for the qualitative subsample of the larger Recovery Finance sample are presented in Table 1. The sample self-identified predominantly as male (64%) and middle aged (M = 43.1, SD = 11.2) Participants self-identified as African American (23%), White (54%), and Hispanic/Latine (27%). Close to half of the participants reported having obtained a high school education or General Educational Development (GED) diploma (45%), and most participants indicated they were “looking for work – unemployed” (77%). Most identified as straight/heterosexual (91%), single (54%), and having children (68%).

**Table 1:** Demographics and behavioral health characteristics of qualitative sub-sample at baseline.

Selected variables collected at baseline	Mean/%	SD/N
Number of participants in the qualitative subsample		22
Gender Identity (self-identified)		
Male	64%	14
Female	36%	8
Average age	43.1	11.2
Race (check all that apply) – self-identified		
American Indian/Alaskan Native	4.5%	1
Asian	4.5%	1
Black or African American	23%	5
White	54%	12
Other	14%	3
Ethnicity: Hispanic/Latine	27%	6
Education:		
Less than High School	23%	5
High School Graduate	27%	6
GED	18%	4
Partial College	23%	5
Associates Degree	9%	2
Sexual Orientation (self-identified):		
Straight/Heterosexual	91%	20
Gay	4.5%	1
Bisexual	4.5%	1
Marital Status (self-identified):		
Single	54%	12
Married/Living in permanent relationship	23%	5
Divorced, separated, widowed	23%	5
Living with someone? Yes	45.5%	10
Employment status:		
Working full-time or part-time	14%	3
Working only temporarily	4.5%	1
Looking for work	77%	17
Other	4.5%	1
Number of months employed last 2 years	6.4	7.6
Do you have children?	68%	15
Have you ever served on active duty in the U.S. armed forces, military reserves or National Guard? Yes	0%	22
Incarceration History		
At what age was your first incarceration?	24.1	10.1
At what age was your most recent incarceration?	38.9	12.2
How many times have you been incarcerated?	5.1	4.2
Are there any legal cases pending for which you could end up in jail or prison during the study? Yes	4.5%	1
Are you on probation or parole? Yes	50%	11
Mental Health		
Age first mental health or emotional problems	15.7	10.8
Currently receiving mental health services? Yes	59%	13
Are you satisfied with your mental health care provider? Yes	92%	12
Hospitalized (ever) for psychiatric reasons? Yes	50%	11
How many times over your life have you been hospitalized for psychiatric reasons?	8.6	11.2
Do you take psychiatric medication for mental health	41%	9
Patient Health Questionnaire (PHQ-9) Sum Score	8.8	7.0
PROMIS Anxiety-8a Sum Score	21.4	9.7
PCL-5 Sum Score	32.5	21.9
Money and Mental Health Average Score	2.1	1.0
Substance Use		
Currently receive Substance Use treatment? Yes	41%	9
Are you satisfied with your SU care? Yes	100%	9
Have you benefitted from a self-help group, such as church group, AA, NA etc.? Yes	73%	16
Have you ever tried to stop using drugs with or without help? Yes	82%	18
Are you currently taking medication to help you with your substance use? (please choose all that apply)		
No	70%	14
Methadone	25%	5
Suboxone	5%	1
Major Life Challenges		
Have you ever experienced homelessness? Yes	86%	19
How many times have you experienced homelessness?	3.3	3.1
Have you experienced childhood physical, verbal, or sexual abuse? Yes	46%	10
Have you ever witnessed physical, verbal, or sexual abuse as a child? Yes	50%	11
ACES Sum Score	5.0	3.0
Wellness		
Overall Wellness (across domains) average	3.3	0.5
Physical wellness average	3.0	0.6
Intellectual wellness average	3.6	0.4
Environmental wellness average	3.6	0.6
Spiritual wellness average	3.5	0.6
Emotional wellness average	3.4	0.6
Financial wellness average	2.4	1.0
Social wellness average	3.4	0.6
Occupational wellness average	3.4	0.7
Social Determinants of Health		
Living situation needs sum (range 1–13)	4.3	1.4
Food needs sum (range 2–7)	5.0	1.6
Transportation needs sum (range 0–2)	1.2	0.5
Safety needs sum (range 4–20)	8.5	4.3
Family and community support needs sum (range 2–9)	5.6	1.6
Education needs (range 0–2)	0.7	0.6
Preventative Services obtained sum (range 0–5)	8.5	7.7
Employment needs (range 1–3)	1.8	0.5
Financial Strain (range 1–3)	2.3	0.7

#### Incarceration history

All participants had histories of legal system involvement. The average age of first incarceration was 24.1 (SD = 10.1), and an average of 5.1 (SD = 4.2) times incarcerated. Half (50%) reported being currently on probation or parole.

#### Mental health and substance use challenges

The sample reported significant mental health challenges: the first onset of mental health problems occurred at an average age of 15.7 (SD = 10.8), 59% were currently receiving mental health services, and 41% were taking psychiatric medication. Nearly half (50%) had a history of psychiatric hospitalization, with an average of 8.6 times lifetime admissions (SD = 11.2). The average PHQ-9 summary score indicated participants reported depression almost reached the “moderate depression - treatment indicated” summary score of 10 (M = 8.8, SD = 7.0). Participants' responses on the PROMIS Anxiety 8a measure had an average sum score of 21.4 (SD = 9.7; T-score of 59.4), which is in the “mild” to “moderate” range for anxiety (healthmeasures.net). Participants’ average sum scores on the PCL-5 (M = 32.5, SD = 21.9) are in the range of probable PTSD (i.e., a score of 31 or higher is indicative of probable PTSD). Participants' responses to the Money and Mental Health measure (M = 2.1, SD = 1.0) indicated that their finances affected their mental health in the “sometimes” to “often” range.

Substance use challenges were also evident: 41% were currently in substance use treatment and 73% had attended a self-help group such as Alcoholics or Narcotics Anonymous. The majority (82%) reported having tried to quit using drugs. Additionally, 30% reported currently taking medication to help with their substance use recovery (see Table 1).

#### Major life challenges

Participants also reported experiencing major life challenges. 86% reported a history of homelessness (mean number of times = 3.3, *SD* = 3.1). Nearly half (46%) reported experiencing childhood physical, verbal, or sexual abuse, and just over half (50%) reported witnessing physical, verbal, or sexual abuse as a child. Participants' responses to the ACES questionnaire (M = 5.0, SD = 3.0) demonstrated participants had on average 5 experiences with childhood trauma, which is above the cutoff score of 4, indicating high risk for serious chronic health conditions, disability, and increased suicide risk in adulthood.

#### Wellness

Participants' responses show their average overall wellness was in the “sometimes true” range for wellness behaviors (M = 3.3). The highest wellness scores were in Environmental and Intellectual wellness (M = 3.6), and the lowest wellness subscale scores were in Financial wellness (M = 2.4) and Physical wellness (M = 3.0).

#### Social determinants of health

Participants endorsed substantial challenges related to their living situation, food needs, transportation needs, safety needs, needing help finding work, wanting help with school or training, feeling lonely/isolated, needing help with day to day tasks, and financial strain (e.g., on average participants reported it being “somewhat” to “very” hard “to pay for the very basics like food, housing medical care, and heating”).

### Qualitative findings: mental health and peer support experiences

Qualitative findings are presented from interviews with 22 people; 15 of whom completed both a baseline and follow-up interview. Demographics of the qualitative sample interviewed are listed in Table 2 (using Aliases and masking potentially identifying data) and are similar to the demographics of our overall sample.

**Table 2:** Demographic information for qualitative subsamples.

Alias	Gender ID	Age	Race	Latine?	Times	MH	SU	Housing status	Randomization Group
Ashley	Male	30–39	Black/AA	No	1–3	Yes	No	Worried	FCS
Monica	Female	30–39	White	Yes	4–6	Yes	Yes	Steady	FCS
Ron	Male	50–59	White	No	10–12	Yes	No	Unsteady	FCS
Lauren	Female	40–49	White	No	1–3	No	Yes	Unsteady	FCS + PS
Darren	Male	60–69	Black/AA	No	10–12	No	Yes	Unsteady	FCS + PS
Andrew	Male	30–39	Asian	No	1–3	Yes	No	Steady	FCS
Travis	Male	40–49	Black/AA	No	7–9	No	No	Steady	FCS + PS
Mandy	Female	50–59	White	Yes	1–3	No	No	Steady	FCS + PS
Roger	Male	50–59	Black/AA	No	1–3	No	No	Steady	FCS
Suzanne	Female	40–49	White	No	1–3	Yes	Yes	Worried	FCS
Maurice	Male	40–49	Black/AA	No	4–6	Yes	Yes	Unsteady	FCS + PS
Nolan	Male	50–59	Multiracial	Yes	1–3	No	No	Unsteady	FCS + PS
Henrietta	Female	40–49	White	No	1–3	No	No	Worried	FCS + PS
Sally	Female	20–29	White	No	13–15	Yes	Yes	Unsteady	FCS + PS
Fred	Male	60–69	White	No	10–12	Yes	No	Worried	FCS + PS
Dawson	Male	20–29	American Indian	Yes	1–3	No	No	Unsteady	FCS + PS
Jaime	Male	30–39	Multiracial	Yes	7–9	Yes	Yes	Steady	FCS
Donald	Male	50–59	White	No	1–3	Yes	No	Worried	FCS + PS
Richard	Male	40–49	White	No	1–3	Yes	No	Worried	FCS + PS
Denise	Female	40–49	White	No	1–3	Yes	Yes	Unsteady	FCS + PS
Beatrice	Female	30–39	White	No	4–6	No	No	Unsteady	FCS + PS
Mateo	Male	30–39	Multiracial	Yes	10–12	Yes	Yes	Worried	FCS + PS

Times – Range of number of times incarcerated; MH - “Are you currently receiving mental health services?”; SU - “Are you currently receiving substance use services?”; Steady = I have a steady place to live.; Worried = I have a place to live today, but I am worried about losing it in the future.; Unsteady = I do not have a steady place to live (I am temporarily staying with others, in a hotel, in a shelter, living outside on the street, on a beach, in a car, abandoned building, bus or train station, or in a park.) FCS = Financial Capability Support, FCS + PS = Financial Capability Support plus Peer Support.

#### Themes

Thematic analysis of interview data with participants revealed the following key themes:

1. Incarceration, Trauma, and Mental Health.
2. Systemic Failings in Mental Health Care in Prison and Post-Release.
3. The Cycle of Financial Strain and Mental Health.
4. The Unique Value of Shared Lived Experience through Peer Support.

##### Theme 1: incarceration, trauma, and mental health

The recovery finance sample included people with diagnosed serious mental illness as well as people who had self-identified mental health problems but no formal diagnosis. Many described having mental health and/or substance use problems prior to incarceration, which in some cases contributed to their arrest. Most narratives revealed trauma related to poverty, abuse or violence as a child or young adult, early experience of mental health or substance use, and/or criminal activity, criminal justice involvement (either arrest or incarceration).

Maurice: “my mental health started because I found one of my friends dead. So, I was dealing with that and never took care of it. I think over time it just accumulated… then it blew up, suicide attempt… being shot, all types of stuff… so I just self-medicated, like trying to get it out of my head, cause it just gets hard, just always thinking about it”

Dawson: “… a woman that’s a [expletive] Christian, letting your son be in the street, sleeping in the parking lot, having nowhere to stay.”

A few people, by contrast, described comfortable and loving upbringings with involvement with the criminal justice system arising due either to serious mental illness or substance use.

Donald: “I had a mental illness, bipolar two… linked to psychosis. I got in a fight with my brother that just wasn't pretty, so I got … in trouble for that. [But as a family] it brought us closer. My parents are married for 54 years, so we're a tight family. They didn't press charges or anything, they knew I was sick … [now] I talk to [my brother] six times a day”

Regardless of mental health problems prior to incarceration, most people said their experience in prison negatively impacted their mental health, with lasting consequences even after release. They spoke of a constellation of issues - the pain of being separated from loved ones, loss of autonomy, lack of access to open spaces or greenery and nature, loneliness, disrespectful treatment by prison staff, an ever-present fear of theft and violence from other people in prison, and anxiety about what life might be like after release. While not everyone mentioned all these things, overall the narratives show how incarceration can damage a person's existing relationships and their ability to form new relationships, and undermine their dignity, sense of self and safety, affecting their ability to manage life after release.

Jaime: “I'm a high security inmate when I go to jail, so I can't see the grass from my window… I felt different. I wear yellow jumper. Everybody else got tan. Made me feel ..not [part of] a community… it made me stick out like a black sheep… that’s some of the reasons why I try to lash out on my body or property due to me feeling bad and the mental health kind of amplifying my thoughts and stuff”.

Mateo: “In jail, you…can't sleep… they got dorms… there’s a bunch of people around you.. You feel, they gonna steal your stuff and you gotta fight, you know? Or if you fight you go to the box when you get out… all your property is gone because they steal it.”

Maurice: Anxiety and the worrying [in prison] … because I didn't have nowhere to go. Worrying about… homelessness, stuff like that”.

Despite the joy and relief of being out of prison, many people described lasting negative mental health effects. While a few said the experience had increased their resilience and ability to manage everyday stresses outside, many more said their mental health had worsened. Some spoke of being unable to shake memories of experiences in prison – as Henrietta said, “*[I still have] racing thoughts and memories of losing everything upon my incarceration and my family being separated… I don't like bright lights anymore because the COs [Correctional Officers] would leave it on as punishment”*. Several spoke of jumpiness, especially if people are behind them, or if they hear keys or sirens – as Roger put it - “*sometimes … I feel like someone's behind me or something. It's not always been like that, where I felt like someone was over my shoulder.”*

Two people, both of whom described significant trauma associated with prison, nonetheless identified aspects they appreciated – namely the structure and routine, and forced separation from unhealthy activities. This tension between prison as a place of trauma, and as a source of structure and a space for someone to change their behavior, points to the dire lack of community-based, non-punitive or carceral options to support positive behavior change.

Ashley: I didn't want to go home; I wanted to stay… in jail. I mean, you know when you're getting three meals, you know when laundry is, you know when you get your commissary, what time mail call is, you know what time you go to church… some people need structure.”

Monica: “after…years of homelessness… the last time I got arrested it was a safe haven… I needed that structure and I felt like everywhere I went [prior] I had the control to leave - even rehabs - so I felt like jail…saved me this time… because the streets were very, very dangerous, the way I was living. I had many altercations where I almost died. So, because I had no control of myself at that time… the last time I went to jail, I believe, I did it on purpose”

##### Theme 2: systemic failings in mental health care in prison and post-release

People reported mixed experiences with mental health treatment while incarcerated. Some experienced forced restraints which made things worse; Monica, who in some ways appreciated the structure of prison (see above) described particularly disturbing treatment.

Monica: “You're not treated like a human being in there… And them not even knowing my background and why I was in there… I was struggling with abuse from men and… drug addiction and homelessness… [there was] no kind of therapy there. They throw you in that turtle suit and put you locked up. You're crazy, that’s it. No one to talk to you and find out why, what’s going on, really, in your head… worst part is: they had me butt naked. I had a tampon in; I had my period. They threw a pad at me…and told me to figure it out. And I'm like, so I had to hold it there. So I felt like a piece of dirt, you know?”

Jaime: “If you get cuffed and like, your mind is not really, you know… sometimes you… misinterpret the intentions and … people get hurt because if you're scared because you don't know what’s going to happen or … you… think you're in danger and you move, they'll hurt you… Sometimes people want attention but then there’s these really like important times that people have to learn how to recognize that people need help”‘

The theme of ‘not being treated like a human’ came up frequently, with prison described as a place where the familiar stigma of mental illness and substance use from life outside prison was intensified without recourse. Monica, Jaime and others acknowledged they had problems that needed to be addressed but were frustrated that no-one was willing to listen to them to be able to understand how to help them. They show that they had insight into what they needed, but the prison system failed to come even close to providing that.

Several people said that they were not given appropriate medication in prison, including support with substance withdrawal. This was interpreted less as a mistake due to misdiagnosis, and more because they were not seen as human or worthy of respect or even, as Sally suggests, life-saving treatment.

Sally: “It’s absolutely awful… it was harder to stay on the medications I needed for like my depression and things…. there’s no support system… [also] I'm on methadone and I was addicted to alcohol at the time and benzos … the only withdrawals you could die from is alcohol and benzos. And they basically did nothing for me. It took days to get my methadone”

By contrast, some said they finally got the right medication or treatment when in prison – Donald said, “I haven't had an episode since before I went to jail…. believe it or not, they got me on the right medication when I was in jail, right off the rip”. Jaime, who had been jailed several times, said that he had done well when in a prison specifically for people with serious mental health issues, but he ended up having to ‘act out’ repeatedly to be moved from other facilities to that one. Once at the ‘appropriate’ facility, he was given the right medications, treated better, and his mental health improved.

Jaime: “first when I got locked up, I was at W_ and I explained to them, hey, I've been to G_ [mental health facility] before… But they would not send me to G_. Even though in G_ I did pretty good and being stable for a couple months at a time and whatnot they wouldn't send me. So, I had to act out… I was acting out bad so they sent me to M_ [another prison] and there I came across two cellies [cell mates] that attacked me….the first time…I didn't really know much about what to do, people was like: go back in the cell, clean up.. and come back out, like don't stop us from being outside.. and then the second time I was like “I had it, I had enough” and I just started.. listening to the voices and like doing the bad stuff like, I was doing real bad stuff.. I was peeing all over the place, hurting my head. I did a hunger strike three days without food, stuff like that. I felt like I needed to do that…. then they sent me to G_ and I was doing good over there.. trying to find my purpose in life”

People's experiences of therapy in prison were mixed – some said it was inadequate, with only very brief ‘*one-two minute*' sessions with a counselor, but several had a better experience with both groups and one-on-one counseling. These experiences contrast sharply with those where people felt they were not being listened to or even being considered human.

Mateo: “ [in federal prison] it’s a therapeutic community… they teach a lot, I mean [inaudible], parenting… stuff like that. I did voluntary groups, AA, NA… stuff like that.”

Nolan: “When I had a mental health problem, I always had somebody to go and speak to in there…it make me feel good that I could go and speak to somebody that gonna understand and lend an open ear to listen to my problem and give me good advices”

Darren: “it helps, the groups and stuff, to prepare you for when you… get out. You know, it does help you talk, you know, talk about what’s hurting you, you know”.

After release, problems with treatment, including themes of disrespect, not being listened to, and not being seen as human, continue. People spoke of being prescribed medications with unacceptable side-effects – for example Denise had stopped taking her anti-depression medication as she cannot not risk being sleepy as she is homeless – “*I was like falling asleep and I can't… I gotta be aware, especially [living] outside”.* Denise continued to use substances which she found to be more effective in helping her manage her situation “*[I use drugs when] I..* *just feeling just hopeless going through just trying to just… wanting to be away for a little bit [starts to cry]. Not to mention having things happen in the past to deal with, which started it.”*.

Feeling ignored by medical providers continues for some – as Sally said, “*the psychiatrist lowered and adjusted my medications without even meeting with me… how do you know what I need without even screening me or talking to me?”* Some described delays in receiving medication due to bureaucratic problems with insurance. Henrietta's story illustrates the multiple challenges of reentry, even when a person has a place to stay after release (she was in a halfway house), exacerbated by delays in receiving medications.

Henrietta: “They were not able to fill my prescriptions, and they kept telling me that my insurance was not active… I wasn't able to get my psych meds until a week and a half of my release… and my Vyvanse… for my ADHD, I did not receive until a month after… Going without was… extremely difficult. I ended up isolating myself. And… when I did leave the house to actually go to an appointment, my anxiety was through the roof because I reside… where I don't know the area. I didn't know anyone. And on top of it, you know, like I said, I was away from the world for over five years. It was very hard to get back into the community, and I still struggle to this day”

While those who mentioned prison-based treatment groups were generally positive, experiences in the community were less so. Several people mentioned compulsory groups or programs they did not find helpful that impeded their search for employment. Some described the groups and programs cynically as part of a check list, more about allowing providers of those services to continue to be employed than actually meeting the needs of people with problems. The themes of disrespect, not being listened to and thus not having one's needs met, continue from prison to the community after release.

Darren: “as far as you trying to get a job.. there’s no time. You know, they want you to do this [group]. They want you to do that [program]. How can you get a job or continue to get money to support yourself? You've got to do all these things.”

Sally: “rehab… was a joke… I was supposed to stay 45 days, but I only stayed like 20, and they just gave me a certificate of accomplishment, because they just wanted to get me out, because they were so tired of me… The groups I just slept through”

Travis: “I know one brother [who] knew people that was working there. So, they pushed him up and he get an apartment, but he had to go to a mental health thing for like a week or two. And he don't need mental health. So, to me it was like: you just going to places to do stuff… is saying it’s a business to me”

A few people had more positive experiences with mental healthcare after prison but emphasized their preference for one-on-one treatment over groups. Challenges included finding a person who they could meet with consistently and develop a relationship with; this is a familiar challenge with public mental healthcare where providers are not easily accessible without intensive case management or medical insurance.

Richard: “I've been going to counseling at [behavioral health services agency] bi-weekly, since about a month after I got out. And I meet with a gentleman and… I just kind of ramble… and it’s gone really well.”

Maurice: “I’m mostly trying to find somebody one-on-one that I'm comfortable talking to, enough to tell my problems. [In] the groups… you don't share as much… before, I was going to [behavioral health services agency] I dealt with this one person for I think it was probably a couple of months. But then they switched [and] once you switch.. you have to deal with [inaudible] over and over. So… I stopped going after that. But if I could actually find someone I could be with for a while and just comfortable talking to them.”

##### Theme 3: the cycle of financial strain and mental health

Many participants described stress related to financial challenges. Several said that worrying about not being able to pay bills, keep their phone on, or help loved ones caused them more anxiety than anything else. Falling behind on bills can have disastrous consequences, such as eviction, utility shut off or losing the use of a phone. Phones are essential – particularly for people with health challenges or required reentry programs who must keep up with appointments and communicate with providers and probation officers. Inability to provide support to loved ones risks damaging important relationships, which are more critical than ever during the reentry period.

Roger: “[when] we get our bills in, I… stress about that. Because I know I gotta make sure those bills get paid. And if we don't have the money.. The bills. That’s the only thing that we're stressing out”

Denise: “my phone has been shut off before… It’s the scariest thing to not be able to have any contact. I mean.. our phone is the only reason we're even like, it’s the only way we could even get anything”

Richard: “Like you always have to go somewhere and call someone and fill out paperwork, even SNAP benefits [food stampes] or whatever, you have to figure out a way to call them. How do you call them if you don't have money to get a phone?

Some mentioned difficulty getting back on disability benefits or food stamps after release from prison. Nolan described the frustration of having to deal with a bureaucracy that doesn't seem to make sense, and in the meantime he was going hungry.

Nolan: “I don't have to worry about the rent. It’s other bills like phones, I don't know how imma keep my cell phone on…and… I'm trying to get on food stamps. They keep asking me for a letter that I'm not working. What letter? How I give you a letter that I am not working, I am NOT working..They keep saying the same thing to me”

People not eligible for benefits have to work to earn income; several spoke about the challenges of finding a job with a criminal record. As mentioned, mandatory attendance at groups can impede the search for work, as can halfway house rules. From the perspective of people coming out of prison, the system appears to contradict itself – on the one hand finding work is highly encouraged as the mark of not just financial stability but also respectability and belonging, on the other hand the barriers to finding work can feel almost insurmountable.

Several said that being unable to find a job pushed them back to selling drugs to earn money, and of course risk being reincarcerated. This cycle of poverty, prison and mental health is perfectly summed up by the words of Travis and Richard, both older men who had long periods of incarceration through their lives.

Travis: “me being outside on the street selling drugs, it really started because… my mother was on welfare and that’s how it was with me coming up… I started selling drugs because I didn't want my mother to - and she was working hard - be giving me money, because she was babysitting. So, I, at a young age thinking, okay, if I'm selling drugs, then my mother don't gotta give me money. So, I use to hide it from her. She didn't know, but when she gave me money, I wouldn't take it. I was like: “Nah ma, save your money”, as a young one. So, as I'm coming up… you get addicted to the drug game… And then that’s all you know. And then [after release] was trying to find a job… for so long. It was so hard. I’ve never been a depressed person. I was trying to do right, so bad and not do wrong. It depressed me. I was trying to get a job, and I started selling drugs [again].”

Richard: “it’s just really tough right, right at the beginning… everything is thrown on you immediately… there’s no safety net… the pressure is ramped up immediately… if you're 27 years old, and you've been in prison for six years, and you come out and all you've ever known is the drug game, and all you've ever known is getting high yourself, all of a sudden you walk out and you're hit with all of these pressures financially. What do you do? You find a way to sell drugs, and you get high to take away the stress…that’s why it’s just such a vicious cycle of people just coming out, going back in, coming out, going back in, coming out, going back in. There’s just no financial support.”

Some people worried about debt and bad credit, fearing its effect on their ability to find an apartment or open a utility account; most landlords require a good credit score, and it can be impossible to open a utility account if you have an unpaid balance.

Denise: “To have that at the back of your head that… something’s lurking, you know, something’s owed. My head is just always going… the gas bill and [electric] bill, even if I were to get a place I couldn't get anything turned on, so… that’s what I worry about”

##### Theme 4: the unique value of shared lived experience through peer support

Several people mentioned the importance of support from people with similar life experiences who could empathize with them, either as friends, or more formally through support groups run by people with relevant lived experience, or one-on-one support. Their narratives illustrate the value of trust, not being judged, not wanting to be patronized, and of being able to check in rather than having to attend a formal appointment. Critically, they spoke of support from people who had managed to move away from negative activities and could walk alongside them on a more positive path.

Monica: “we used to use [together] on the streets, and she was doing all right. She was in there [prison] and she brought me food off her commissary… when my family wouldn't reach out and nobody would write me a letter, at least I had positive people…. [others] are not on the same path as me, so I have to leave them behind. Not everyone’s gonna go where you're going. So, yep, I got a positive network now… a network that I can run to that I am treated fairly like a human being, a person not judged for who I was.”.” Roger: “I get more out of B_’s [Recovery Finance peer supporter who runs a peer support group] group than I do out of AA. That’s what keeps me going… I love it. I'm happy… we see..how much impact she had on us…And she’s real.. it’s even better… She’s been there.”

Sally: “I go to… women’s groups… twice a week… I've been real close with some of the women there… I consider them.. my friends because I don't have any family and some of them have no family either. So, for holidays, some of us would, you know, just go to one of their houses, you know, and just cook there and spend it with each other because we had nobody else or nowhere else to go just so we wouldn't feel, you know, so depressed and alone”

Travis: “they been wanting me to go speak to somebody [a counselor], and I don't want to. It’s like, to me, I can handle it. I could do it. If I, if I really get that bad, I need to talk to somebody, I can go talk to [the Recovery Finance peer supporter]”

People also valued being able to use their own lived experience to support others experiencing situations like theirs.

Nolan: “I also would like to be somebody’s advisor, because I've been here on TikTok live a lot… we always help each other there. We got a little crew. Somebody going through something, we always speak to them. So, I would like do that, too, speak to people”

Monica: “I definitely love [working] in the hospital. I get to touch some people’s souls with my story. I get to make people feel better, even when they don't feel better. And not look at them as that, “what’s the matter with them?” I think I became more compassionate because I walked them shoes”

Jaime: “… re-entering and trying to move forward without overwhelming yourself and going back to jail without support or contacts that could actually help you improve yourself… it’s hard ….I think about it like this, if I could help myself be successful, I could help other people.. to help themselves because I know exactly the steps.”

Henrietta: “I became a recovery coach during my incarceration. [It] has actually changed my life… I found a purpose for myself in this world when I was told at the age of five that I wasn’t supposed to be here, I wasn’t wanted. So being a part of [recovery community], it’s very good for my recovery to remain in recovery. It’s like a whole different world there. And the family that I ended up developing is amazing”

### Reflections from recovery finance FPS peer supporters

To ground the above themes in practice, the project's Forensic Peer Support specialists provided written reflections on their work. The following anonymized case narratives, presented in the supporters' own words, exemplify the application of peer support within the Recovery Finance intervention addressing the challenges identified above.

#### Example 1

I connected with Maxwell through the Peer Support/Financial Recovery program. We began by building rapport and focusing on his primary goal of finding full-time employment. He was getting work through day labor but couldn't secure a stable job. Recently released after a long incarceration, he also wanted guidance on reentry, opening a bank account, and building credit. We met weekly to formulate strategies. He achieved his job goal after I suggested he apply at [a grocery store]—he was hired there. Because there's a bank inside the store, he was also able to open a checking account and get a secured credit card, hitting all his financial goals. For reentry needs, I linked him with a nonprofit that provides everything from basic needs to mental health resources. He used this support, which made our peer work smoother. Maxwell lives with his girlfriend and their kids, so we often talked about the struggles of relationships after prison. It's a rule of thumb not to give advice but just to listen to matters of the heart, so that's what I did. I listened. He and I did come to a consensus that being incarcerated for a lengthy period of time has a way of allowing comfort in being alone.

Thus, it takes time to get used to sharing space with people all day, every day.

#### Example 2

I worked with Adrian. Connecting was simple because he had one clear goal: to find a way to stay “clean” from opioid use. Through phone calls and home visits, we discussed what that could look like. He lives with and provides daily care for his elderly mother, so we first focused on financial wellness, finding a job to help support her care. Since jobs weren't immediate, I connected him with food resources. After that, our connection relaxed; he began to accept me as someone there for support. He opened up about his challenges with opioids and past attempts to get “clean”. He wasn't interested in traditional 12-step programs, so I told him about a faith-based program that offers sobriety through worship, charity, and community. I expressed that he had the right to choose to leave this program when and if he desired, yet it initially requires an 18-month commitment during which he will be provided with food, clothing, and housing at no cost to him. I took him to meet the pastor which runs the program. While Adrian hasn't joined the program yet, he has accumulated many sober days. He continues to care for his mother, ensuring she takes her medication, and maintains that strong bond.

#### Example 3

My six months working with Jay were very interesting because he came from the federal prison where one of my family members was at the time of my first meeting with him. I noticed that he was sizing me up and started questioning me. I answered each question until he was satisfied. After the very next meeting he became relaxed and open to start trusting me a little more. It was also unique because he came from a gang called (deleted). At first, I was hesitant to work with a gang member, and I had concerns for folks seeing me with him in the community. He wanted me to know that his gang were not robbing and killing folks. They were guys who helped the community and not the type to take from it. For example, they had fund raising to help kids get book bags.

I think that anyone who comes from jail automatically comes home with some kind of mental health issues or trauma, so I thought that would be an easy way to connect with him. I truly listened to him because I wanted to know about who really was sitting in front of me. During the time that I met with him, I noticed that he kept talking about drinking alcohol when he had free time to do so. I talked to him about some of my journey concerning getting high off the drugs I had used. He couldn't believe the amount of years that I self-medicated. With him using alcohol, I know that the feds would pack him back in jail for it. We talked about it; I just suggested that he can put down a few less drinks before his counselor catches on. He really did not drink until his mandatory group that he had to do on a daily basis was done. After the fourth time meeting him, he got back in trouble, and he was afraid to go back to court. I also think that he picked up a petty charge to put him back in jail. I told him to go because he had been down that road, and he knew that eventually he would see the streets again. I talked to him until the day he got packed back in and went back to jail. He wanted to go on the run; however, I suggested to him that it wasn't worth it. I met him in person around 5–6 times, and he called at least 3–4 times a week until they took him to jail. This experience really hit close to home, with my family members walking through the same jail. It brought up a lot of emotions for me, watching another Black male going back to jail and watching the self- medication, hopelessness, emptiness, not good enough for society, anger etc. I can go on and on – it's not a good feeling when you can do anything but give folks hope. What a connection and I glad that I tried and it went well. I do miss the conversation because he gave me hope for my family member who is still in the system.

#### Example 4

How was it connecting with Mike in the six months that I have been working with him? I thought it was amazing. I built a relationship with him before I met him in person. I had already talked to him about ten times - each time that we had scheduled a time to meet something always went wrong, so I felt connected already. He basically mentioned his son that was in and out of his life, and how diabetes was attacking his toes. Each time that we talked I suggested for him to make an appointment so that he can take care of his foot. Finally, he made an appointment after I mentioned to him about my family member getting half his leg cut off. He took care of his foot and got one of his toes cut off and felt a lot better. I met him at Dunkin Donuts, and we broke bread together. He also mentioned that his son came by the [homeless shelter] to reconnect with his Dad. I truly think that if folks have someone to assist when dealing with issues, things go along much smoother than when you don't have someone. Most folks would love to be a part of something bigger than themselves when they have someone to walk side by side with them. His relationship with himself and his son was powerful and for him to add me to his journey was amazing. His responsibility to take care of his health was powerful. The last thing was just his commitment to continue to pick up the phone for me – that meant a whole lot. If I had to do it again, I would not hesitate to jump on board.

## Discussion

This paper reports on mental health and peer support aspects of qualitative narratives from 22 individuals, a sub-sample of a larger sample (*n* = 234) of participants of a research intervention seeking to help people with mental health challenges who were formerly incarcerated with their finances. Our findings reveal the persistent and interrelated challenges that formerly incarcerated individuals experience well beyond their release. From a community health perspective, the findings reveal systemic and structural gaps in the continuity of care, social supports, mental health and substance use recovery and economic security.

Many participants had mental health issues before prison; all said that being incarcerated negatively affected their mental health – sometimes dramatically so - due to being separated from loved ones, loss of autonomy, loneliness, being treated disrespectfully by prison staff, experiences and fear of theft and violence and anxiety about their life after release. Most had a poor experience of mental healthcare offered in prison, though a few mentioned positive aspects even when their overall experience was not good. These people pointed to the structure and routine afforded by prison life, being removed from negative environments they had lived in prior and access to therapy and groups.

Mixed experiences of mental health care continue after release, with many experiencing inconsistent and inappropriate treatment with too few options to meet diverse needs, compounded for some by challenges with insurance. The period immediately after release is extremely challenging, and it is critical that people receive support to navigate the relative freedom of being out, which can be immediately compromised by siloed programs that contradict each other and seem nonsensical, and inability to make ends meet. Indeed, while most spoke of release as a relief from the strictures and stresses of prison life, they described continued mental health stressors, particularly lack of access to good mental health care and lack of social support, as well as financial challenges due to difficulties finding employment and attaining disability benefits, and experiences of homelessness. These stressors can result in return to criminal activity and using substances, creating a cycle of recidivism.

Another significant finding of this study is the value of social support in the form of family, partners, friends, and professional peers in the post-incarceration period. Peer support was described as more accessible and trustworthy than formal services, especially in the early stages of reentry. Not only did people appreciate peer support themselves, they also recognized that their lived experience enabled them to offer valuable support to others in situations like theirs. This type of support - being treated with respect, being listened to and taken seriously - counters the themes running through experiences in prison and in society after release. The accounts of the FSPs show how they are able to build relationships with people that are based on respect, listening, and taking the person seriously as a human being.

### Implications for future practice and research

The findings of this study have several important research and practice implications for community health practice when working with those involved with the carceral system. There is an urgent need to improve conditions in prison, particularly for people with mental illness and substance use disorders. This may involve training for prison staff, and measures to ensure that those who are particularly vulnerable are protected and not stigmatized. It is critical to improve and build the capacity of diversion programs to reduce the numbers of people moving through the carceral system. Where appropriate specialized correctional facilities exists, systems must be in place to ensure that those who need such specialized support can access it. We should also learn from those aspects of incarceration that some people spoke of positively, offering people non-or minimally carceral options that provide structure, ability to separate from negative environments and access supportive treatment.

The widely varying experiences we heard about mental health care and treatment in prison could point to idiosyncrasies of the individuals we spoke to but are more likely to reflect varying quality of care across different facilities. High quality mental health care should be available at every facility, regardless of a person's length of stay. We should maximize the opportunity while someone is incarcerated to provide needed high quality treatment and support, and work to develop a continuum of such care – both one-on-one and group-based - through the transition to the community after release. Guaranteed and seamless access to health insurance must be part of this, as well as intensive case management. The cynicism expressed by some of our participants point to a need for more person-centered, trauma- informed care and treatment that visibly consider each person's particular situation and needs rather than imposing standardized requirements on everyone.

Much of what we are recommending may require additional resources – that is money – be invested in creating person-centered carceral and mental health systems that gives people the support they need to maximize opportunities for recovery and successful reentry.

Participant narratives starkly highlighted the deeper structural connections between poverty, trauma and incarceration. Cycles of repeated behaviors are rooted in a system that for far too many people offers nothing but bad choices. Until we have a political economy that ensures everyone can be affordably and safely housed, and that does not criminalize homelessness and addiction, the cycle will continue. In the short-term, measures can be taken that address the financial aspects of criminal behavior (e.g., selling drugs), such as offering more opportunities for job training and placement leading up to and after release, as well as guidance and support to maximize financial stability in that critical time period. This could involve financial counseling at the time of sentencing and before release, along with support retrieving lost IDs and links to safe and affordable bank accounts. Providing an adequate basic income after release to ease the transition to financial stability may be needed.

Our findings also emphasize the critical role of social connections – be those informal with friends and family, or with professional peer supporters. This can be challenging for people with histories of substance use and criminal activity who often want to separate from people who they feel have a negative influence on them; supporting people to discover new connections and community is important. We should also continue to increase the availability of well-trained and properly remunerated peers who can support such connections. Taken together, these findings highlight the interconnected nature of mental health, financial instability, and social support during reentry. Importantly, they also point to critical implications for how peer support can be leveraged within financial wellness interventions to address these intersecting challenges.

### Implications for peer-supported financial wellness interventions

Findings from this study point to the critical role of peer support in financial wellness interventions for individuals returning from incarceration. Participants described financial stress not only as a practical challenge, but as a central driver of anxiety, instability, and risk for returning to previous survival strategies. These results indicate that financial wellness cannot be appropriately addressed solely through traditional financial coaching, particularly for people navigating complex reentry barriers.

Peer support addresses this gap. Peers can connect with participants in ways that traditional providers often cannot, including trust building more quickly, ‘normalizing’ financial struggles, and encouraging open conversations about debt, income instability, and survival behaviors that may otherwise be stigmatized or hidden. Peer supporters can also help individuals address issues in real time, connect to resources, and navigate fragmented systems. Peer support can be initiated immediately upon release, a time marked by vulnerability and a higher risk of recidivism. Participants' experiences suggest that even with supports, financial pressures can quickly escalate, undermining both the mental health and the recovery journeys of individuals. Early peer engagement can provide continuity, relational support, and practical guidance at a time when individuals are often disconnected from formal services.

These findings point to the need to have integrated models in which peer support is embedded within financial wellness and reentry services. Such models should prioritize rapid linkage to peer support post-release, ongoing relationship-based engagement, and coordinated connections to financial resources, employment opportunities, and social services.

### Recommendations

Based on the findings of this study, we recommend:

1. Expanding peer roles to include financial navigation and support may be a key strategy for addressing the intertwined challenges of mental health, poverty, and recidivism.
2. Expanding mental health and substance use treatment within correctional facilities that include early and periodic assessment and evidence-based approaches; discharge planning that connects individuals to community providers with secured appointments and services upon release.
3. Improved partnerships and collaboration between correctional facilities, community providers, and social services to support holistic re-entry and secure social service benefits and healthcare benefits.
4. Improved re-entry focused and community programs that offer coordinated care that address mental health, substance use, housing, employment, finances and vocational services.
5. Investing in peer and social support programs with sustainable funding and incentives for those who provide support.
6. Eliminating systemic barriers to employment and housing by advocating policy and legislative changes that contribute to the discrimination of individuals formerly incarcerated or with criminal records.

### Limitations and future directions

The generalizability of the findings are limited by a single geographic location for data collection, the small sample of 22 participants, limited demographic diversity, and non-random (i.e., purposive sampling), although the purposive sampling was intended to ensure representation of participants from both intervention groups and with different baseline level of financial strain. Limitations to the quantitative data include self-report or recall bias and a cross- sectional design. Nonetheless, this study still provides rich insight into the experiences of those formerly incarcerated who are impacted by mental health issues and financial security and how peer support can help improve financial health.

Future research should include samples from other geographic locations and settings within longitudinal studies designed to better understand change in participants' circumstances over time and to evaluate peer-supported financial interventions through the integration of qualitative and quantitative outcomes. Also, the perspectives and experiences from community providers that include mental health services should be explored to identify the systemic barriers to providing quality services to this vulnerable population.

In summary, the findings from this study starkly highlight the deeper structural connections between poverty, trauma and incarceration and demonstrate how cycles of repeated behaviors are rooted in a system that for far too many people offers nothing but bad choices. Peer support, in particular, offers the opportunity for healing from the carceral system where, in peer support relationships, people are treated with respect, listened to, and taken seriously. The findings of this study suggest important research and practice implications for community health practice when working with those involved with the carceral system. The findings also support specific policy recommendations to offer treatment for behavioral health challenges and facilitate successful societal re-entry that addresses employment, financial, and vocational challenges. Peer support has the potential to intervene to support and address these important social determinants of health, in particular, mental health and financial health.

## Data Availability

The raw data supporting the conclusions of this article will be made available by the authors, without undue reservation.
