# Supplementary material for: Peer support to address mental health, social determinants of health, financial wellness, and reentry navigation
Source: Front Health Serv. 2026 Jun 16;6:1788228. doi: 10.3389/frhs.2026.1788228 (PMC13315229; doi:10.3389/frhs.2026.1788228)
Supplement: Supplementary file 1 [file Table1.docx]

**Recovery Finance Qualitative Interview Guide – 6-MONTH FOLLOW-UP**

**BASELINE**

As you know, we are interested in learning about the financial challenges faced by people who have been incarcerated and have mental health challenges. Your stories are important in understanding what types of financial problems you have, how those problems affect your life, and what we can do to try to help avoid these financial problems.

**Part 1: Who are you?**

**To begin, I would like to know more about you, who you are as a person and anything you may want to share with me about your life.**

1. Tell me about yourself.

*(Probe: This can include where you grew up, your favorite activities in your spare time, and how you see your role in your family or community.)*

1. How are things going for you lately?

**Part 2: Incarceration**

1. I would like to ask you about your experiences with the criminal justice system. Please tell me about your first experience with the criminal justice system and how it affected you.

*Probe: How old were you? What happened? How did it impact you as a person?*

1. What about the time you spent in prison? Tell me something about that, and how it affected you?

*Probe: how did it affect your mental health, your relationship and role within your family?*

1. What happened with your finances while you were incarcerated?

*Probe: How did money play a role in your life while incarcerated? How did you meet your day-to-day needs and any obligations you had outside of incarceration, such as family contributions, child support, or debt and savings? Did you receive any financial support, i.e. family/friends contributed to your commissary, where you able to work in prison, etc.?*

**Part 3: Finances & Mental health post-incarceration**

1. Tell me something about how your life has changed since you were released from prison. *Probe: What is your current living situation? Have you been able to reconnect with friends and family?*
2. What about your mental health? How do you think your mental health was affected during incarceration, after your release? How are you doing today?

*Probe: What are some of your concerns? Were you receiving any mental health services before your incarceration? Did you receive any services during and after*?

1. Let’s talk about your financial situation since you were released. Tell me about your financial history since you were released, and how things are today.

*Probe: if you had a bank account before going to prison, could you still access it afterwards? Did you owe anyone money from when you were inside? What happened to your credit score? How are you meeting your financial obligations today? Have you been able to get an income since you were released? Does your income cover your expenses? Can you afford to pay rent? Did you have to depend on anyone else or borrow money to get buy or meet your basic needs? Did you experience any type of identity theft while you were incarcerated?*

1. When you think about your financial situation, now and in the past, how do you think your upbringing and your childhood may have affected it?
   1. *Probe (just in case) Who contributed to the household income? How did your family manage emergency expenses, such as funeral costs or supporting relatives in crisis? What did you learn about money growing up? What role did you play in your family's financial obligations, both as a child and now?*
2. What are your hopes for your future regarding your financial health and your mental health? What are you looking forward to learning from your time in this project?

*Probe: Ask the person if they have met with Rose, the financial coach from Winning Ways. If they were randomized into peer support ask if they have met with a peer. If they have met the coach/peer, ask how that has gone. If not, ask why.*

1. Is there anything else you would like to share with me? Anything I did not cover that you want to include?
